# A *de novo* monoclonal immunoglobulin deposition disease in a kidney transplant recipient: a case report

**DOI:** 10.1186/1752-1947-8-205

**Published:** 2014-06-18

**Authors:** Benjamin Savenkoff, Perrine Aubertin, Marc Ladriere, Cyril Hulin, Jacqueline Champigneulle, Luc Frimat

**Affiliations:** 1 CHU Nancy-Brabois – Service de Néphrologie, Nancy, France; 2 Hôpital Robert Schuman Metz – Service de Néphrologie, Metz, France; 3 CHU Nancy-Brabois – Service d’Hématologie, Nancy, France; 4 CHU Nancy-Brabois – Service d’Anatomie Pathologique, Nancy, France

**Keywords:** Light chain deposition disease (LCDD), Myeloma, Nephrotic syndrome, Post-transplant lymphoproliferative disorder (PTLD), Renal graft

## Abstract

**Introduction:**

Myeloma following kidney transplantation is a rare entity. It can be divided into two groups: relapse of a previous myeloma and *de novo* myeloma. Some of these myelomas can be complicated by a monoclonal immunoglobulin deposition disease, which is even less common. Less than ten cases of monoclonal immunoglobulin deposition disease after renal graft have been reported in the literature. The treatment of these patients is not well codified.

**Case presentation:**

We report the case of a 43-year-old white European man who received a renal transplant for a nephropathy of unknown etiology and developed a nephrotic syndrome with kidney failure at 2-years follow-up. We diagnosed a *de novo* monoclonal immunoglobulin deposition disease associated with a kappa light chain multiple myeloma, which is a very uncommon presentation for this disease. Three risk factors were identified in this patient: Epstein–Barr virus reactivation with cytomegalovirus co-infection; intensified immunosuppressive therapy during two previous rejection episodes; and human leukocyte antigen-B mismatches. Chemotherapy treatment and decrease in the immunosuppressive therapy were followed by remission and slight improvement of renal function. A relapse occurred 8 months later and his renal function worsened rapidly requiring hemodialysis. He died from septic shock 4 years after the diagnosis of monoclonal immunoglobulin deposition disease.

**Conclusions:**

This rare case of post-transplant lymphoproliferative disorder with an uncommon presentation illustrates the fact that treatment in such a situation is very difficult to manage because of a small number of patients reported and a lack of information on this disease. There are no guidelines, especially concerning the immunosuppressive therapy management.

## Introduction

Myeloma following kidney transplantation is a rare entity. Approximately 30 cases have been described in the literature. The disease either occurs in the rare situation of recurrent myeloma – initial degradation of native renal function caused by a myeloma followed by complete remission allowing kidney transplantation – or in a context of a post-transplant lymphoproliferative disorder (PTLD) [1], that is, *de novo* myeloma. Renal monoclonal immunoglobulins deposits can complicate the hematological disease in many different ways (cast nephropathy, amyloid light chain amyloidosis, non-amyloid fibrillary glomerulonephritis, immunotactoid glomerulonephritis and so on), all of them resulting in renal function impairment. One of these renal monoclonal immunoglobulins deposits pathologies is called monoclonal immunoglobulin deposition disease (MIDD), also known as Randall disease [2]. *De novo* MIDD is very exceptional in kidney transplant recipients, less than ten cases have been reported in the literature [3-6]. We present the case of a patient who underwent kidney transplantation for nephropathy of unknown etiology, then developed *de novo* MIDD associated with kappa light chain multiple myeloma, revealed by a nephrotic syndrome and kidney failure.

## Case presentation

A 43-year-old white European man was referred to our unit with an early end-stage renal failure disclosed by asthenia and malignant hypertension. At admission, laboratory tests showed: serum creatinine 7.0mg/dL; blood urea nitrogen 200mg/dL; proteinuria 1.5g/24 hours (albuminuria 75%); negativity of Bence-Jones proteinuria; microscopic hematuria; normal immunology tests; normal serum protein electrophoresis (absence of monoclonal gammopathy and no hypogammaglobulinemia). His renal arteries were normal at the Doppler ultrasound. We tried to perform a renal biopsy but we failed because we only got fat tissue. He required peritoneal dialysis.

Eleven months later, a cadaveric renal graft was transplanted (pediatric donor who died from hemorrhagic stroke; cytomegalovirus (CMV), status: donor+/recipient+). Epstein–Barr virus (EBV), herpes simplex virus, and toxoplasmosis serologies were positive; other serologies were negative. The human leukocyte antigen (HLA) compatibility was: A2-A29/B45-B51/DR1-DR14/DQ5-DQ5 (patient); A2-A24/B7-B60/DR13-DR15/DQ5-DQ6 (donor). A serum protein electrophoresis was performed in the patient at this time and was normal (absence of monoclonal gammopathy and no hypogammaglobulinemia).

The initial immunosuppressive treatment included antilymphocyte serum. The post-transplantation period was uneventful with diuresis on day 1 and serum creatinine 1.7mg/dL (Modification of Diet in Renal Disease, MDRD, formula: 48mL/minute/m^2^) at discharge. The immunosuppressive treatment at discharge was: prednisone (20mg daily); ciclosporin (175mg twice a day); mycophenolate (1000mg twice a day).

Two months after transplantation, CMV reactivation required oral treatment with valganciclovir and a decrease in the immunosuppressive doses: prednisone (5mg daily); ciclosporin 110mg twice a day; mycophenolate (1000mg twice a day).

Seven months after transplantation, he was hospitalized for renal function decline. A graft biopsy was performed and showed borderline acute rejection; immunofluorescence for light and heavy immunoglobulin chains was negative. Treatment with methylprednisolone (five intravenous boluses at decreasing doses) was followed by a decrease in serum creatinine which returned to its initial level (1.3mg/dL).

He was hospitalized for the same reasons 18 months after transplantation. The graft biopsy again showed borderline acute rejection; immunofluorescence for light and heavy immunoglobulin chains was again negative. Treatment was again methylprednisolone (five intravenous boluses at decreasing doses), with the same results.

His general status declined 22 months after transplantation. At admission, an examination revealed rapid weight loss, long-standing productive cough, and herpes zoster with a Ramsay Hunt syndrome. His renal function was altered: serum creatinine 2.4mg/dL (MDRD 31mL/minute/1.73m^2^); proteinuria 7g/24 hours (6g albumin). The three main hypotheses explaining this *de novo* nephrotic syndrome in this kidney transplant recipient were, by reported frequency [7,8]: allograft glomerulopathy; recurrent baseline nephropathy; and *de novo* glomerulopathy.

The immunosuppressive treatment was diminished because of a herpes zoster flare-up (prednisone 5mg twice a day, ciclosporin 100mg twice a day, mycophenolate 500mg twice a day). He also received oral valaciclovir.

A new graft biopsy was performed and revealed severe glomerular involvement with flocculocapsular synechiae, scleronodular mesangial thickenings involving 13 of 30 glomeruli analyzed, a few zones of segmental and focal hyalinosis, peritubular thickening and interstitial fibrosis with tubular atrophy estimated at 50%. The tubules were free of myelomatous casts. Immunofluorescence disclosed monotypic kappa light chain immunoglobulin deposits in the glomerular mesangium and peritubular spaces. Congo red staining was negative. However, electron microscopic examination was not performed because we did not expect to find monoclonal immunoglobulin deposits and thus we fixated no specific sample in glutaraldehyde. Considering this biopsy, the diagnosis of MIDD was retained [2]. Serum protein electrophoresis revealed a monoclonal gammopathy with a kappa light chain monoclonal peak at 964mg/L (free light chains assay Freelite^®^ produced by Binding Site^®^), kappa/lambda ratio at 102, hypogammaglobulinemia, and hypoalbuminemia at 25g/L related to the nephrotic syndrome. A bone marrow aspiration showed 16% atypical plasma cells (high nucleus to cytoplasm ratio, mature chromatin, nuclear inclusions) corresponding to a kappa monotypic clonal population. These results confirmed the diagnosis of MIDD secondary to kappa light chain multiple myeloma without extrarenal event. Polymerase chain reaction on total blood samples was positive for EBV at the time of diagnosis, signaling systemic EBV reactivation. However no direct EBV research was performed on the bone marrow examination at this moment. We considered that both myeloma and MIDD occurred *de novo* in this patient because there was no evidence of plasma cell dyscrasia before, and the two previous graft biopsies showed no immunoglobulin deposits.

He was transferred to the hematology unit and treated for this PTLD by chemotherapy (bortezomib plus dexamethasone). For the immunosuppressive treatment, mycophenolate was discontinued progressively and ciclosporin was pursued, as was prednisone on days without dexamethasone.

The treatment was followed by remission of his myeloma with a decrease in the serum light chains and a slight improvement in renal function; serum creatinine improved from 3.5 to 2.7mg/dL.

He had a relapse with an elevated light chains level 32 months after transplantation. A second chemotherapy regimen was given (bortezomib-cyclophosphamide-dexamethasone) with decreased immunosuppression (ciclosporin reduced to 75mg twice a day).

Nevertheless his renal function worsened rapidly and he required hemodialysis 3 years after transplantation. He received a third line of chemotherapy (lenalidomid plus dexamethasone) for a second relapse of his myeloma 54 months after transplantation.

**Figure 1:**
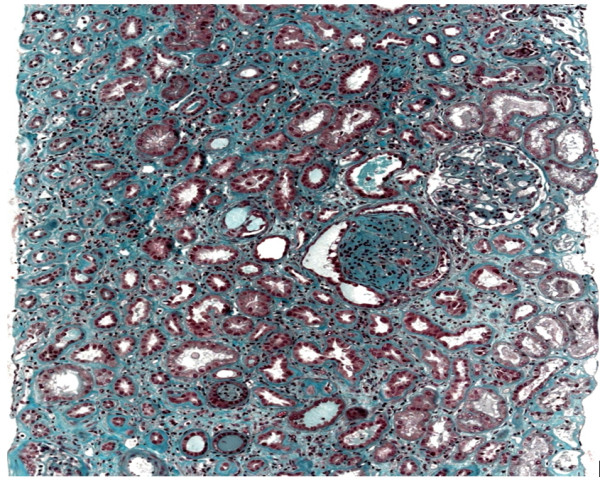
Standard stain, low magnification: interstitial fibrosis with tubular atrophy and presence of a sclerous glomerulus with flocculocapsular synechiae.

**Figure 2:**
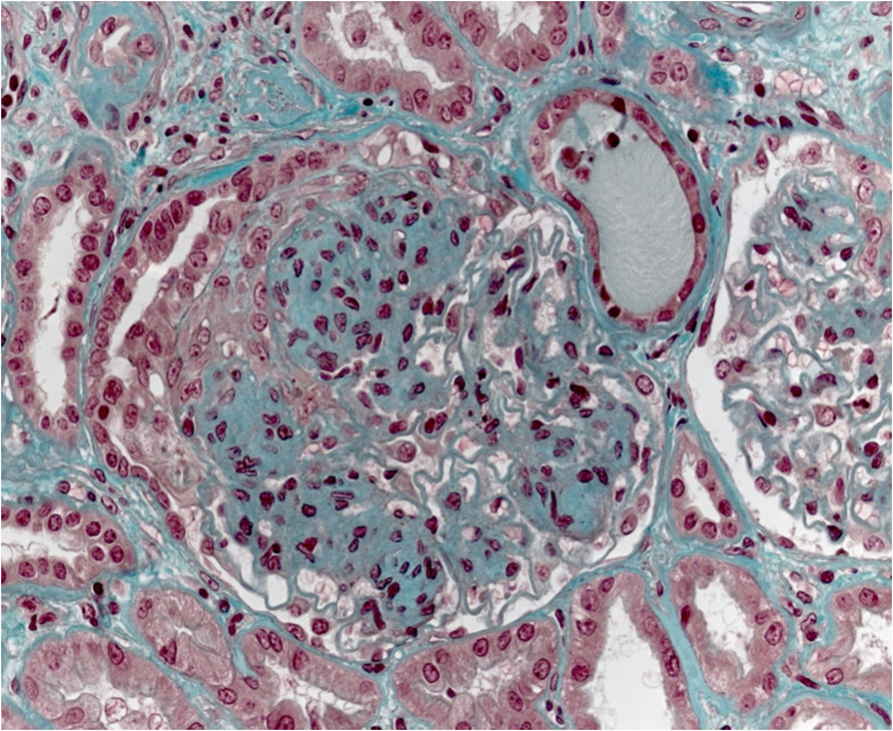
**Standard stain, higher magnification than in Figure**1**: nodular glomerulosclerosis and presence of a protein cylinder in one tube (related to the severe nephrotic syndrome).**

**Figure 3:**
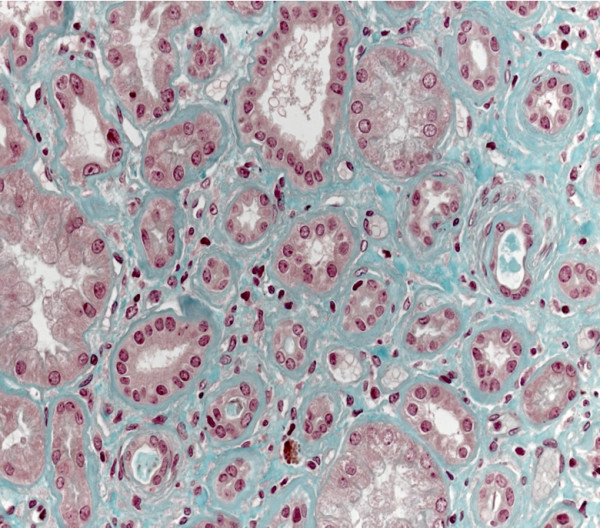
**Standard stain, same magnification as in Figure**2**: major baseline membrane thickening.**

**Figure 4:**
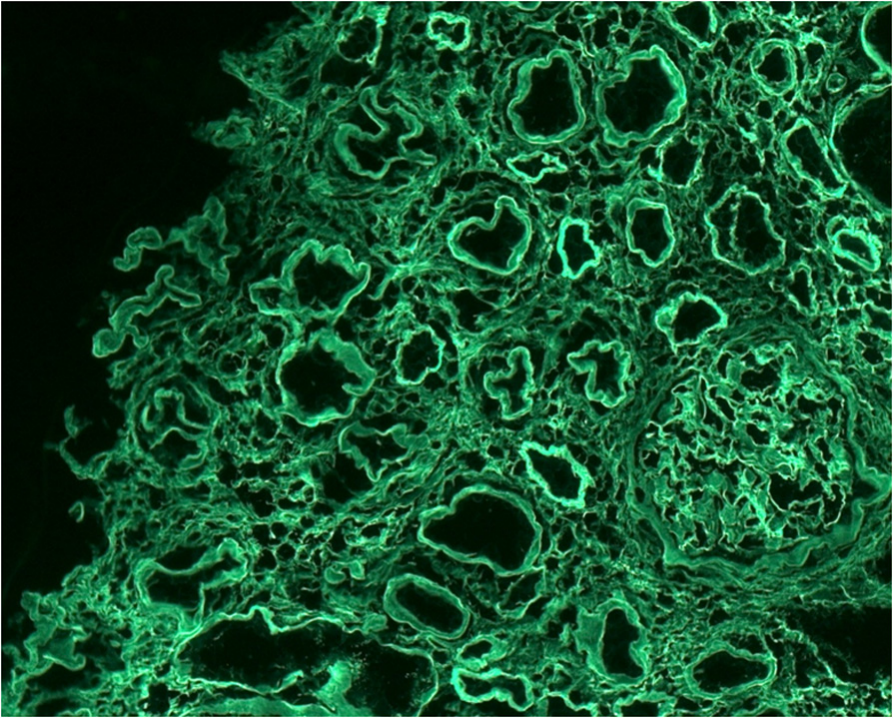
Immunofluorescence with kappa light chains: monotypic kappa light chain deposits in the glomeruli and in the tubular baseline membranes.

**Figure 5:**
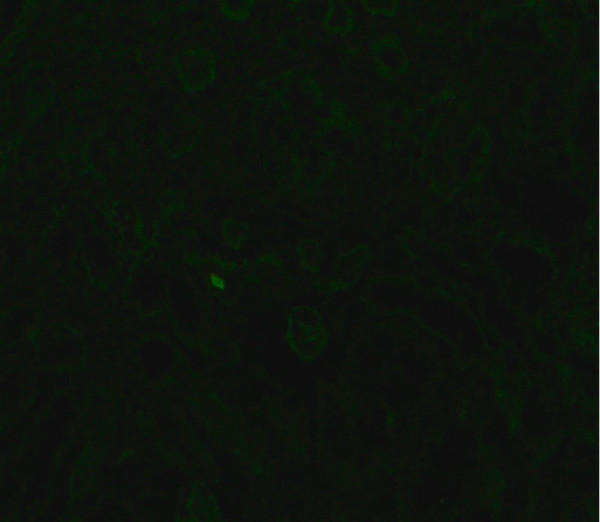
Immunofluorescence with lambda light chains: absence of deposits (negative control).

It is important to notice that we performed a follow-up bone marrow aspiration at this period that confirmed the myeloma relapse and showed 10% of tumoral monoclonal plasmocytes. The *in situ* hybridization did not permit us to find EBV in these tumoral cells.He finally died from septic shock in a context of post-chemotherapy medullary aplasia at the age of 50, 4 years after myeloma diagnosis and approximately 6 years after transplantation (Figures 1, 2, 3, 4 and 5).

## Discussion

Post-transplant myeloma is a rare disease and can occur in the situation of recurrent myeloma (diagnosed or not prior to transplantation) or can occur *de novo*. Several problems are emphasized by this case report.

First of all, it can be difficult to assert the *de novo* character of myeloma because sometimes a kidney biopsy was not performed or was not contributive before transplantation. Moreover, as shown by Balamuthusamy *et al.* in their case report [6] it can be a latent pre-transplant form of myeloma or even a simple monoclonal gammopathy of undetermined significance that may transform into an aggressive myeloma after transplantation in the context of immunosuppressive therapy. In our case we considered that both myeloma and MIDD occurred *de novo* after the renal transplantation because we had strong arguments to assert that there was no plasma cell dyscrasia and no evidence of monoclonal immunoglobulin in the patient’s blood or urine when he was referred to our unit for the first time. In addition, two graft biopsies were performed when his renal graft function worsened and there was no evidence of monoclonal immunoglobulin deposits. As shown by Taneda *et al.* in their article [5] there is a gradual progression of kappa light chain deposition in the renal allograft and these deposits are usually present within the kidney a long time before it becomes clinically symptomatic.

The *de novo* form of myeloma is a B-cell PTLD, like diffuse large B-cell lymphoma [1]. The incidence is low, accounting for 3 to 13% of all PTLDs depending on the series [9,10]. Patients are older compared to the lymphoma group [9]. Risk factors are still not clear but they seem to be the same as for all PTLDs:

– primary EBV infection or EBV reactivation (with CMV and hepatitis C virus co-infections identified as risk cofactors) [9,11];

– major immunosuppressive treatment; in particular, inductive antilymphocyte serum and long-term anti-calcineurin drugs whereas other immunosuppressive agents are less associated with this risk or even have a protective effect as observed in certain studies with antimetabolites and anti-mammalian target of rapamycin drugs such as sirolimus) [9,12,13];

HLA incompatibility, especially for HLA-B (direct effect by creation of a latent antigen conflict with secretion of lymphoproliferative cytokines and indirect effect by causing graft rejection requiring more intense immunosuppression) [14-16].

Our patient presented all the above risk factors: EBV reactivation with CMV co-infection; intensified immunosuppressive therapy during both rejection episodes; HLA mismatches, particularly for HLA-B.

These risk factors should be systematically searched among patients with *de novo* plasma cell dyscrasia, which is rarely emphasized in the literature.

Regarding the management strategy for these patients, a systematic screening for EBV in tumor plasma cells using an *in situ* hybridization technique would be advisable because the majority of these hematological diseases are virus related [17]. If viral reactivation is documented, an antiviral treatment can be proposed; in particular, ganciclovir because Foscavir^®^ (foscarnet) is nephrotoxic [18,19]. In our case, direct EBV tests were not performed at the time of diagnosis and no antiviral treatment was given.

A third problem concerning *de novo* myeloma or MIDD after transplantation is that the treatment strategy is not well codified.

It would be probably advisable to diminish the immunosuppressive treatment because several studies have demonstrated that PTLDs regress after decreasing anti-rejection treatments [12]. No clear guidelines are available, but it would appear reasonable to reduce the anti-calcineurin dose first because several studies have linked these agents with a higher relative risk of PTLD compared with other treatments [9,13,20]. In our patient, the mycophenolate dose was reduced first and ciclosporin was maintained at the same dose, which illustrates the heterogeneous management strategy, especially concerning the immunosuppressive treatment. This immunosuppressive treatment strategy is not well clarified in the literature dealing with *de novo* plasma cell dyscrasias. Usually, immunosuppressive therapy is rapidly stopped when graft function worsens because of a very high infection risk compared to the benefit of the treatment.

New therapeutic options are being studied for unresponsive forms of the disease, for example plasma exchange [4], immunotherapy [21] or injection of anti-EBV cytotoxic lymphocytes [22].

## Conclusions

Currently, the development of myeloma in a kidney transplant recipient raises several problems related to the lack of specific therapeutic guidelines, the rapid decline of renal function (direct effect of myeloma, risk of rejection due to reduced immunosuppressive treatment, chemotherapy-related nephrotoxicity), and the major risk of infection (immunosuppression, myeloma-related hypogammaglobulinemia, vascular access for dialysis).

In this case report we outline and discuss all of the above problems. First of all, the disease occurred as a *de novo* glomerulopathy with renal graft failure and nephrotic syndrome. We tried to gather strong arguments to assert the *de novo* character of MIDD and myeloma which is not always easy. Then, our patient presented the three main risk factors for PTLD which are rarely mentioned in the literature. This raises also the question of EBV reactivation treatment. We illustrated the fact that management strategy is not homogeneous and that clinicians, nephrologists or hematologists, have to face many problems when treating these patients. Last but not least, this case report emphasizes the very bad prognosis of this kind of PTLD that often leads to end-stage renal graft failure and severe infectious complications.

To conclude, a systematic report study of *de novo* myelomas or plasma cell dyscrasias among kidney transplant recipients would be useful to better define the characteristic features of these patients and to organize large-scale trials to determine a more evidence-based management strategy.

## Consent

Written informed consent was obtained from the patient for publication of this case report and accompanying images. A copy of the written consent is available for review by the Editor-in-Chief of this journal.

## Abbreviations

CMV: Cytomegalovirus; EBV: Epstein–Barr virus; HLA: Human leukocyte antigen; MDRD: Modification of Diet in Renal Disease (formula used for the glomerular filtration rate estimation); MIDD: Monoclonal immunoglobulin deposition disease; PTLD: Post-transplant lymphoproliferative disorder.

## Competing interests

The authors declare that they have no competing interests.

## Authors’ contributions

BS (corresponding author) collected, analyzed and interpreted the patient’s data, looked for references from the literature and wrote the manuscript. PA collected, analyzed and interpreted the patient’s data and wrote the abstract. ML and CH were the clinicians in charge of the patient and provided intellectual content. JC performed all the histological examinations. LF was the investigator of the manuscript project and provided intellectual content. All authors read and approved the final manuscript.
